# The Boston Puerto Rican Health Study, a longitudinal cohort study on health disparities in Puerto Rican adults: challenges and opportunities

**DOI:** 10.1186/1471-2458-10-107

**Published:** 2010-03-01

**Authors:** Katherine L Tucker, Josiemer Mattei, Sabrina E Noel, Bridgette M Collado, Jackie Mendez, Jason Nelson, John Griffith, Jose M Ordovas, Luis M Falcon

**Affiliations:** 1 USDA Human Nutrition Research Center on Aging, Tufts University, Boston, Massachusetts, USA; 2 Northeastern University, Boston, Massachusetts, USA; 3 Friedman School of Nutrition Science and Policy, Tufts University, Boston, Massachusetts, USA; 4 Tufts Medical Center, Boston, Massachusetts, USA

## Abstract

**Background:**

The Boston Puerto Rican Health Study is an ongoing longitudinal cohort study designed to examine the role of psychosocial stress on presence and development of allostatic load and health outcomes in Puerto Ricans, and potential modification by nutritional status, genetic variation, and social support.

**Methods:**

Self-identified Puerto Ricans, aged 45-75 years and residing in the Boston, MA metro area, were recruited through door-to-door enumeration and community approaches. Participants completed a comprehensive set of questionnaires and tests. Blood, urine and salivary samples were extracted for biomarker and genetic analysis. Measurements are repeated at a two-year follow-up.

**Results:**

A total of 1500 eligible participants completed baseline measurements, with nearly 80% two-year follow-up retention. The majority of the cohort is female (70%), and many have less than 8^th ^grade education (48%), and fall below the poverty level (59%). Baseline prevalence of health conditions is high for this age range: considerable physical (26%) and cognitive (7%) impairment, obesity (57%), type 2 diabetes (40%), hypertension (69%), arthritis (50%) and depressive symptomatology (60%).

**Conclusions:**

The enrollment of minority groups presents unique challenges. This report highlights approaches to working with difficult to reach populations, and describes some of the health issues and needs of Puerto Rican older adults. These results may inform future studies and interventions aiming to improve the health of this and similar communities.

## Background

Racial and ethnic disparities in prevalence and incidence of chronic conditions are an important problem in the United States (US). Their investigation can contribute to progress toward their elimination and improved understanding of the etiology of diseases [1-5]. Reduction of existing racial and ethnic health disparities is a primary concern for ethical and economic reasons. Without improvements, as minority populations grow and age so will the burden on the health care system. Hispanics currently represent 14.8% of the US population and are projected to increase to almost 25% by the year 2050 [6].

Most epidemiologic research on Hispanics has focused on Mexican Americans, due to their majority as a subgroup. However, evidence suggests that health outcomes vary significantly by Hispanic ethnic subgroup; geographical and ethnic variation have been reported for the prevalence of type 2 diabetes among elderly participants in the US, South America and the Caribbean [7]. There is evidence of variation in management of diabetes among Latino subgroups, which suggests that grouping Hispanics into one category may obscure important differences [8].

Puerto Ricans are the second largest Hispanic subgroup in the US [9]. They report the worst health status and highest prevalence of several acute and chronic medical conditions, when compared with non-Hispanic whites (NHW) and other Hispanic subgroups [10]. National data show that 21% of older Puerto Ricans reported having an activity limitation, compared with 15% of Cuban and Mexican Americans [11]. Puerto Rican elders living in Massachusetts show significantly greater prevalence of physical disability, type 2 diabetes, depression and other chronic conditions than NHW living in the same neighborhoods [12-18].

There is a paucity of knowledge regarding the health status and behaviors of Puerto Rican adults living in the US. Moreover, little is known about strategies for recruitment and retention when conducting research with this population. Therefore, the goal for this report is to describe the challenges and opportunities for recruitment and assessment of a cohort of Puerto Rican adults living in the Boston, MA area, as well as their baseline characteristics. The Boston Puerto Rican Health Study (BPRHS) is funded as part of the Centers for Population Health and Health Disparities, a special initiative of the National Institutes of Health to foster trans-disciplinary and multilevel research to improve understanding of health disparities in the US [19].

## Methods

### Study design

The BPRHS is an ongoing longitudinal study that aims to examine the role of psychosocial stress on the presence and development of allostatic load (physiological dysregulation) and health outcomes such as depressive symptomatology, cognitive impairment, functional limitations, and metabolic conditions in Puerto Ricans. Further investigation includes the potential modification of these associations by nutritional status, especially for dietary fat, B vitamins and antioxidants, by genetic variation, and by sources and type of social and community support. The study was approved by the Institutional Review Board at Tufts Medical Center and Northeastern University. All participants provided written informed consent.

### Recruitment

Participants are recruited from the Greater Boston area using door-to-door enumeration and community approaches. Data from the 2000 Census was used to identify census tracks with at least 25 Puerto Rican adults, ages 45-75 years. Within these, randomly selected census blocks with 10 or more Hispanics, ages 45-75 years, were enumerated door-to-door. Blocks were visited at least three--and up to six--times, on different days of the week, including weekends, and at varying times of day, including evenings. Households with at least one eligible adult were identified. One participant per qualified household was randomly invited to participate.

Similar to other studies [20-22], multiple recruitment strategies were used. In addition to the door to door enumeration, participants were identified by random approach during community festivals/fairs and events sponsored by local community organizations, through referrals, and through calls to the study office from flyers distributed at community locations, or from radio or television spots about the study. The community partner, La Alianza Hispana, is a non-profit organization serving Latinos in the Boston area. They provided advice and assistance with the recruitment and retention efforts, and continue to serve as a liaison with the participants.

Eligible participants must be of self-identified Puerto Rican descent, able to answer questions in English or Spanish, ages 45-75 years, and living in the Boston, MA metropolitan area at the time of the study. Individuals who were unable to answer questions due to serious health conditions, planned to move away from the area within two years, or who had a low Mini Mental State Examination (MMSE) score (≤ 10) were excluded. Interviews were scheduled after the initial screening contact. Participants were provided with a written reminder of the interview date, and were called 1-2 days prior to the interview to remind them. For those who cancel, rescheduling was attempted at least five times, after which participants were considered de-facto refusals.

### Data collection

Baseline questionnaires and tests were administered by trained, bilingual interviewers in the participant's home. After obtaining informed consent, neuropsychological tests were conducted to identify participants that may need assistance from a proxy, or be excluded due to low MMSE score. Those who qualified completed the rest of the interview. Participants were given a 12-hour urine collection cup and two saliva tubes, with procedures for collection and instructions on fasting for the next day's blood draw. A certified phlebotomist returned to collect the samples and draw blood in the home.

All interviewers were thoroughly trained by experienced staff to administer the questionnaires and to perform measurements following procedures from National Health and Nutrition Examination Survey (NHANES) II [23] and the MacArthur Studies of Successful Aging [24]. Retraining and review sessions, including checks on scoring of tests and scales, were conducted periodically. Each new interviewer was required to observe several interviews by experienced interviewers and to practice repeatedly before collecting data. Completed interviews were self- and peer-reviewed prior to database entry.

#### General background characteristics

Participants provided information on age, education level, household income, migration, acculturation, employment history, family size and food security. These questionnaires were designed based on NHANES III [25,26], the Hispanic Health and Nutrition Examination Survey [27,28] and the National Health Interview Survey Supplement on Aging [29]. Poverty status was computed using the poverty thresholds released each year by the US Census Bureau [30]. Each participant's total annual household income was compared to the threshold based on the age of the head of household, participant's family size, and year of interview. If total household income was less than this threshold, the participant was considered to live in poverty.

Acculturation was captured by a set of questions on language use in a number of everyday activities [31,32]. This language-based questionnaire was adapted from the Bi-dimensional Acculturation Scale for Hispanics (BAS), which focuses on language preference in various settings [33]. This allows for the possibility that acculturation entails the acquisition of American cultural traits without displacing Hispanic ones. The scale yields two scores which rank participant's acculturation in the Hispanic domain and the non-Hispanic domain; a value of 100% represents a fully acculturated participant in the non-Hispanic domain who speaks fluent English. A psychological acculturation scale that focuses on psychological attachment to either culture was also administered. The scale was validated with three different samples of Puerto Ricans from the greater Boston area [34].

#### Health and health behaviors

Participants were asked to self-report whether they have been diagnosed with a specific list of chronic conditions. Detailed information on prescription and over-the-counter medications was collected by asking participants to show the bottles for medications they currently take. Participants provided health insurance information and self-rated their health status and satisfaction with health care practices. Frequency, history, and type of alcohol consumption and smoking were assessed. Current physical activity was captured using a modified Paffenbarger questionnaire of the Harvard Alumni Activity Survey [35,36], which was effectively tested in an elderly Puerto Rican population [14]. A physical activity score was calculated as the sum of hours spent on typical 24-hour activities (heavy, moderate, light, or sedentary activity, and sleeping) multiplied by weighing factors that parallel the rate of oxygen consumption associated with each activity.

#### Anthropometric and blood pressure measurements

Standing height, knee height, weight, and waist and hip circumferences were measured in duplicate [23,37]. Body mass index (BMI) was calculated using weight (Kg) divided by height (m) squared. Systolic and diastolic blood pressures were measured in duplicate, at three time points during the interview. The second and third readings were averaged. Detailed methodology is included in Additional File 1.

#### Physical disability

Participants were asked to report difficulty performing daily activities, with modified Katz Activities of Daily Living (ADL) and Instrumental Activities of Daily Living (IADL) scales [38]. The twelve ADL and six IADL items have been used effectively in a previous cohort of elderly Puerto Ricans [12]. Additional physical performance tests, which measure balance, gait, chair stands, foot taps and manual ability, were completed, following tests used in the MacArthur Studies of Successful Aging [39].

#### Cognitive function

A comprehensive neuropsychological examination assessed specific impairments in cognitive functioning. Tests were selected based on evidence of validity in Spanish-speaking populations, and to evaluate higher cognitive functioning to minimize floor effects. They include the MMSE for general function [40], the word list learning test [41] for verbal memory, the Stroop [41] for mental processing speed, digit span [41] for working memory, verbal fluency [41] for executive function with language, and clock drawing [42] and figure copying [43] for visuospatial function. Cognitive impairment categories, as defined by MMSE scores, were adjusted for educational level, as this correction optimizes the test [44]. For all levels, considerable impairment was defined as a score of 11 through 17; mild impairment includes score of 18 through 21 for participants with middle school, 18-23 for those who completed high school, and 18-24 for those with college or graduate education. Higher scores than those cutoff values by educational attainment were deemed as having no impairment.

#### Depressive symptomatology, stress and support scales

Depressive and anxiety symptoms were assessed using the Center for Epidemiology Studies Depression (CESD) Scale [45-47]. The CESD has shown consistency and validity in older adults [48]. It has also been used with Hispanics [47], including Puerto Ricans [12], with good reliability. The Spanish versions of the Life Events Questionnaire [49,50] and the Norbeck Social Support Questionnaire [51] were used to assess life and psychosocial stress. The Perceived Stress Scale measures the degree to which one's life is viewed as stressful [52], with higher values representing higher perceived stress. The scale has been satisfactorily tested in other Spanish-speaking groups [53,54]. A second stress scale, developed through qualitative interviews with a subset of participants, was also administered in the second wave of interviews.

#### Dietary assessment

Dietary intake was assessed using a semi-quantitative food-frequency questionnaire (FFQ) with 126 items, adapted and validated for this population [55]. The FFQ, based on the National Cancer Institute-Block FFQ format, was revised to include appropriate foods and portion sizes, and was shown to capture intakes reported in 24-hour recalls more accurately than the original questionnaire, both in total nutrient estimates and in ranking of individuals [55]. This FFQ has been validated against plasma carotenoids [56], vitamin E [57] and vitamin B12 [58] in Hispanics aged ≥ 60 years. Those with energy intakes < 600 or > 4800 kilocalories and/or > 10 questions blank on the FFQ were excluded from dietary analyses.

#### Biological measures

Detailed methodology is included in Additional File 1. Blood samples were analyzed for complete blood counts, creatinine, albumin, plasma lipids, total carotenoids, vitamin C, folate, vitamin B12, pyridoxal-5'-phosphate, homocysteine, methylmalonic acid, C-reactive protein, glucose, insulin, glycosylated hemoglobin, blood urea nitrogen, and dehydroepiandrosterone sulfate. A 12-hour urine sample was analyzed for cortisol, creatinine, epinephrine and norepinephrine. Saliva samples were used for measurement of salivary cortisol.

#### Definition of medical conditions

Diabetes status was defined as fasting plasma glucose ≥ 126 mg/dL or use of medication [59]. Hypertension was defined as blood pressure ≥ 140/90 mmHg or use of medication [60]. BMI was used to classify weight status, with BMI 25-29.9 as overweight, 30-39.9 as obese class I and II, and ≥ 40 as extremely obese [61]. Waist circumference > 102 cm in men or > 88 cm in women was defined as high [61]. Depressive symptomatology was defined as CESD score ≥ 16 [62].

#### DNA isolation and genotyping

DNA extraction and genotyping methods have been previously described [63]. Briefly, genomic DNA was isolated from blood using QIAamp DNA Blood mini kit (Qiagen, Hilden, Germany). Genetic polymorphisms were genotyped with Applied Biosystems TaqMan SNP genotyping system [64]. Genotyping error rate was < 1%, as estimated by internal quality control and independent, external laboratories. Genes and polymorphisms were selected by bioinformatics assessment, based on previously reported associations or knowledge of their function in known biological mechanisms. Over 140 autosomal, diallelic polymorphisms, from 35 different genes have been genotyped.

### Two-year follow-up

Two-year follow-up is ongoing at time of writing. All subjects are contacted and asked to participate in a follow-up visit after two years, to repeat all questionnaires and measurements. Participants are called every six months to update contact information (at which point they update a life events inventory questionnaire), and receive holiday and birthday cards and occasional newsletters with updates on the study. At baseline, participants were asked to provide the names of two contacts who could locate them if they moved. These persons are contacted if the participant cannot be reached directly after several attempts. When participants cannot be contacted by phone, letters are sent requesting that they call the study. When there is no response, a staff member visits the home. Awareness of the study in the general community is fostered by participating in community events, attending meetings of community agencies to present information on the study, encouraging articles in both Spanish and main newspapers, and participating in media appearances.

### Statistical analysis

Statistical analyses for this report were completed using the SAS System for Windows (version 9.1, SAS Institute, Inc, Cary, NC). Descriptive analyses to examine differences between sex and age groups (45-59 and 60-75 years) were performed using chi-square analyses for categorical variables and t-tests for continuous variables with normal distributions. Fisher's exact tests were used for variables with an expected cell count of less than five. *P *values were calculated and a significance level of < 0.05 was used. All tests were two-sided.

## Results

### Participant recruitment

**Figure 1:**
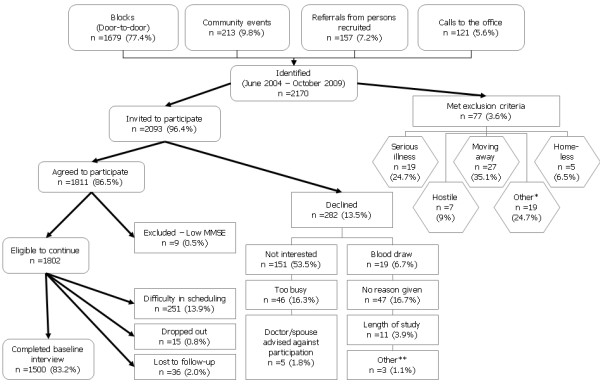
**Flowchart of study participant recruitment for the Boston Puerto Rican Health Study**. * Various reasons including: not Puerto Rican; spouse in study. ** Various reasons including: currently busy, upcoming vacation or surgery, and houseguests, health problems or illness.

The majority of participants were identified through door-to-door enumeration (77.4%), with the rest recruited through community events (9.8%), referrals (7.2%), and calls to the study office (5.6%) (Figure 1). From June 2004 to October 2009, a total of 2,170 Puerto Rican age-qualifying adults were identified. Of these, 77 met the exclusion criteria, and 2,093 were invited to participate. From those invited, 1,811 (86.5%) agreed to be interviewed. Primary reasons for declining included not being interested in the study, too busy, and refusal of blood draw. Those who declined were more likely to be older (58.4 versus 56.7 years, data not shown) and had lived on the US for more years (32.9 versus 28.8 years, data not shown). No significant differences in sex, language spoken or birthplace were observed between those participating and those declining. Among those agreeing to participate, nine participants were excluded due to low MMSE score (0.5%), 15 (0.8%) dropped out, 36 (2.0%) were lost before interview, and 251 (13.9%) were never interviewed due to persistent unavailability. Therefore, 1,500 of 1,802 eligible participants who initially agreed to participate (83.2%), or of 2,084 eligible participants who were initially invited (72.0%), completed the baseline interviews. Results presented in this report include 1,357 participants with completed and cleaned baseline interviews at the time of writing.

Those recruited through door-to-door enumeration, relative to community events or calls in response to advertising the study, were significantly older, more likely to be women, have lower education, less acculturated, and less likely to be employed than those recruited by community approaches (data not shown). They did not differ by poverty status, BMI, or prevalence of type 2 diabetes. Based on year 2000 Census tract data, these recruitment strategies included access to 78% of the Puerto Ricans, aged 45-75 years, living in the Boston area towns with participants in the study. When limited to towns with 5 or more participants, coverage was close to 96% for the door-to-door enumeration approach.

Once recruited, completion of interviews was time-consuming and expensive. Appointments needed to be rescheduled approximately 40% of the time, usually because the participant was not home at the scheduled time, or cancelled at the last minute. Among the most common reasons for cancellations were participant's failure to remember the interview appointment, a conflicting medical appointment, illness or death of a family member, and reported illness by the participant.

### Characteristics of baseline participants

The majority of the sample was female (70%). Mean ages were 57.2 years for men and 57.9 years for women. Older Puerto Rican adults (60-75 years) reported fewer years of education than younger adults (45-59 years); over 60% of older adults had < 8^th ^grade education (Table 1). Approximately 50% of adults in all age categories fell below the poverty level, with older women more likely to be living in poverty. Only one-third of men and one-quarter of women in the younger group, and even fewer older adults, were employed at the time of the study. Residential stability was 50% or more for all groups except for younger men (35.1%).

**Table 1:** Selected baseline characteristics for participants of the Boston Puerto Rican Health Study.

Characteristic^a^	Men (N = 401)		Women (N = 956)	
	
	Ages 45-59 (N = 253)	Ages 60-75 (N = 148)	Ages 45-59 (N = 587)	Ages 60-75 (N = 369)
Age (% within sex)	63.1	36.9	61.4	38.6
Education (≤ 8th grade)	34.8**	61.0	39.3**	65.5
Below the poverty level	49.6*	52.5*	58.2**	68.1
Currently working^b^	32.3**	15.1*	22.6**	7.4
Residential stability^c^	35.1*^;^**	62.6	51.5**	58.9
Living alone	36.3*	41.5	26.6**	48.6
Length of residence in US	32.3 (11.1)**	42.2 (10.8)*	32.1 (11.7)**	36.0 (12.9)
Language-based acculturation^d^	32.5(23.7)*^;^**	24.1 (21.2)*	26.7 (22.7)**	14.4 (17.6)
Psychological acculturation^e^	19.2 (7.4)**	17.3 (6.2)	19.0 (6.8)**	16.8 (6.1)

*P < 0.05, between sex, within age category

**P < 0.05, between age categories, within sex

^a ^Reported as percent or mean (SD), except when noted

^b ^Response limited to participants who have held a paid job for more than 3 months

^c ^Defined as percent of participants with a length of residence ≥ 5 years in the same residence

^d ^Percent of English language use (0-100%)

^e ^Scale of 10-50, ranging from less acculturated to more acculturated

The participants in this Puerto Rican cohort had lived in the US for over three decades, on average; of those aged 60-75 years, men had lived in the US longer than women (42.2 versus 36.0 years). Language-based acculturation scores were significantly lower in the older than younger age group for both men and women. In the younger age group, men were significantly more acculturated than women. Psychological acculturation score showed similar patterns as the language-based scale by age, but men and women had comparable scores.

To evaluate life events and social support, participants were asked to identify important persons in their life and to indicate their perception of how these important persons could support them emotionally or assist them in time of need. Significant differences by sex were found among the younger cohort in the size of the social networks and in the perception of availability of emotional and functional support (Table 2). Younger women reported larger networks, but also a lower perception of existing emotional and functional support from these networks than did men in the same age category. These differences were not found among the older cohort, suggesting that some of the earlier differences may be related to life cycle events like marriage, work, and the availability of kin. Women's social networks included a larger number of blood relatives than the social networks of men (3.7 versus 3.2, respectively). Differences between men and women in the presence of friends, other type of relatives, and others in their networks were not significant (data not shown). Reported sources of social support in this population were limited to close family and friends, with few social contacts, on average, beyond the latter two categories.

**Table 2:** Characteristics of social networks in the Boston Puerto Rican Health Study.

Characteristic (Mean)	Men		Women	
	
	Ages 45-59	Ages 60-75	Ages 45-59	Ages 60-75
Size of social network	5.1*	5.5	5.7	5.8
Average emotional support from social network	14.3*	13.9	13.7	14.1
Average assistance from social network	6.3*^;^**	5.7	5.7	5.7

*P < 0.05; between sex, within age category

**P < 0.05; between age categories, within sex

### Health, health behaviors and chronic conditions

Significantly more men than women in both age groups were overweight, while a greater percent of all women fell into the extreme obesity category (Table 3). Close to 80% of women, in both age groups, had abdominal obesity. Overall, participants were relatively inactive; fewer than 12% of men and 4% of women participated in moderate or vigorous activity. Men were more likely to smoke cigarettes or be heavy drinkers than women. About one in five participants used multivitamins, with no significant differences in use by sex or age group. Men consumed significantly more energy and dietary fiber, but less carbohydrate, vitamin B6 and potassium than women across both age groups (Table 4). Participants in the older age category had lower energy intake, and consumed less fat but more carbohydrate than younger participants.

**Table 3:** Health behaviors and anthropometric characteristics for participants of the Boston Puerto Rican Health Study.

Characteristic (%)		Men		Women	
		
		Ages 45-59	Ages 60-75	Ages 45-59	Ages 60-75
Abdominal obesity^a^		43.0*	45.6*	79.7	84.1
BMI^b^	*Overweight*	46.6*	47.5*	30.4***	30.4
	*Obesity (Class I and II)*	49.5	49.2	51.2	57.1
	*Extreme Obesity (Class III)*	3.9	3.4	18.5	12.6
Physical activity	*Sedentary*	36.7*	46.9*	39.8***	61.4
	*Light activity*	52.2	44.9	56.3	37.8
	*Moderate & vigorous activity*	11.2	8.2	3.9	0.8
Current smoker^c^		38.3*^;^**	27.6*	25.7**	12.8
Non-drinker		48.2*	52.5*	59.0***	75.9
Current moderate drinker^d^		35.5	36.4	36.7	22.2
Current heavy drinker^d^		16.3	11.2	4.3	1.9
Multivitamin use		18.5	21.2	18.2	20.4

*P < 0.05; between sex, within age category

**P < 0.05; between age categories, within sex

***P < 0.05; overall between age categories, within sex

^a ^Waist circumference > 102 cm in men; > 88 cm in women

^b ^Defined as weight (kg)/height (m)^2^; overweight = 25-29.9, obesity class I and II = 30-39.9, and extreme obesity (class III) ≥ 40.

^c ^Significant differences determined for current versus past and never

^d ^Moderate drinker defined as ≤ 1 drink/day in females or ≤ 2 drinks/day in males. Heavy drinker defined as > 6 drinks during one day of drinking, or > 1 drink/day in females or > 2 drinks/day in males.

**Table 4:** Dietary profile of participants of the Boston Puerto Rican Health Study.

Nutrient^a^	Men		Women	
	
	Ages 45-59	Ages 60-75	Ages 45-59	Ages 60-75
Energy intake (kcal)	2541 (899)*^,^**	2264 (842)*	2125 (896)**	1880 (825)
*Macronutrients^b^*				
Total fat	33.7 (5.6)*^,^**	31.2 (5.9)	32.3 (5.4)**	30.6 (5.8)
Saturated fat	10.0 (2.5)*^,^**	9.2 (2.4)	9.7 (2.2)**	9.2 (2.4)
Monounsaturated fat	11.7 (2.1)*^,^**	10.7 (2.0)	11.2 (2.1)**	10.5 (2.2)
Polyunsaturated fat	9.0 (2.1)	8.7 (2.0)	8.7 (2.1)**	8.3 (2.1)
Protein	16.6 (2.6)	16.6 (3.2)	17.0 (3.4)	17.1 (3.1)
Carbohydrates	48.6 (7.4)*^,^**	51.2 (7.6)*	51.3 (7.3)**	53.5 (8.0)
Fiber (g/day)	22.9 (8.7)*	23.1 (9.4)*	20.0 (8.8)	19.7 (8.5)
*Vitamins^c^*				
Vitamin B12 (μg)	9.8 (0.4)	8.1 (0.5)	8.9 (0.3)	8.1 (0.3)
Vitamin B6 (mg)	2.2 (0.04)*	2.1 (0.04)*	2.4 (0.02)	2.2 (0.02)
Vitamin D (μg)	5.4 (0.2)	4.9 (0.2)	5.2 (0.1)	5.2 (0.1)
Vitamin E (IU)	15.5 (0.8)	12.2 (0.3)*	15.8 (0.4)	13.3 (0.2)
Folate (μg)	495.5 (9.2)	482.3 (11.2)	505.8 (5.7)	475.6 (6.8)
*Minerals^c^*				
Calcium (mg)	905.5 (23.9)	810.9 (31.3)*	930.2 (14.9)	925.2 (18.9)
Magnesium (mg)	325.5 (5.1)	307.3 (5.5)	337.2 (3.2)	317.6 (3.3)
Potassium (mg)	3111.8 (44.3)*	3010.2 (56.6)*	3288.7 (27.7)	3159.4 (34.3)

*P < 0.05, between sex, within age category

**P < 0.05, between age categories, within sex

^a ^Reported as mean (SD), unless otherwise noted, of food intake, not including supplements

^b ^Reported as percent of energy unless otherwise noted

^c ^Reported as mean (SE) per day and adjusted for energy intake

A quarter of women aged 45-59 years and over a third aged 60-75 years reported considerable physical impairment (Table 5). Cognitive impairment was significantly higher in women than in men in the older age category; considerable impairment was more prevalent in the older than the younger group. The majority of participants self-reported their health status as fair, but more women reported poor health status. Fifty-one percent of women and 45% of men aged 60-75 years had type 2 diabetes (Table 6). More than three-quarters of all older participants had hypertension. A higher proportion of younger than older participants had depressive symptomatology, but prevalence was high for all groups. Self-report of cardiovascular disease was significantly higher for the older group of Puerto Ricans; there were no significant differences by sex. Approximately 70% of older women self-reported diagnosis of arthritis.

**Table 5:** Physical and cognitive function and self-rated health in the Boston Puerto Rican Health Study.

Characteristic (%)	Men		Women	
	
	Ages 45-59	Ages 60-75	Ages 45-59	Ages 60-75
Physical impairment^a^				
*No impairment*	45.0*	38.8*	27.3**	19.6
*Some impairment*	37.1	46.3	48.1	43.2
*Considerable impairment*	17.9	15.0	24.6	37.2
Cognitive impairment^b^				
*No impairment*	78.5**	74.0*	73.4**	61.4
*Mild impairment*	17.5	15.1	23.2	26.6
*Considerable impairment*	4.0	11.0	3.4	12.0
Self-reported health status				
*Very good or excellent*	14.7*	15.7*	11.1**	5.7
*Good*	27.9	24.5	18.6	11.7
*Fair*	49.0	49.7	56.3	66.0
*Poor*	8.4	10.2	13.8	16.6

*P < 0.05, overall between sex, within age category

**P < 0.05 overall between age categories, within sex

^a ^Defined as Activities of Daily Living score of zero (none), 1-5 (some) or ≥ 6 (considerable)

^b ^Defined based on MMSE score and educational level. Considerable impairment = 11-17 for all levels; mild impairment = 18 through 21 for middle school, 23 for high school, and 24 for college or graduate school; scores above those cutoff values by educational level are considered no impairment.

**Table 6:** Chronic health conditions for participants of the Boston Puerto Rican Health Study.

Health Outcomes (%)	Men		Women	
	
	Ages 45-59	Ages 60-75	Ages 45-59	Ages 60-75
Diabetes^a^	37.2	45.0	33.9*	50.7
Hypertension^b^	64.2*	76.6	58.7*	83.7
Depressive symptomatology^c^	50.0**	43.8**	68.1*	58.6
Cardiovascular disease^d^	17.1*	27.2	15.6*	25.1
Arthritis^d^	27.9*^;^**	46.3	48.6*^;^**	69.2

*P < 0.05, between age categories, within sex

**P < 0.05 between sex, within age category

^a ^Defined as fasting plasma glucose ≥ 126 mg/dl or medication use

^b ^Defined as ≥ 140/90 mmHg or medication use

^c ^Defined as a CESD score ≥ 16

^d ^Cardiovascular disease (heart disease and heart attack) and arthritis are self-reported

### Retention

At the point of this writing, 951 (78.7%) two-year follow-up visits have been completed, from the 1,209 participants who have reached their follow-up scheduling time point (data not shown). Of non-completing participants (258), 85 (7.0%) dropped out, 52 (4.3%) were lost to follow-up, and 17 (1.4) were deceased. The remaining 104 participants are being actively rescheduled due to frequent change of residence within the city, circular migration to and from Puerto Rico, and disconnected telephones. Although exact two-year timing of follow-up is difficult, participants are continuously being contacted. Increased participation and retention has been attained throughout the course of the study, as it becomes better known in the community. The remaining 291 baseline participants will be scheduled at their appropriate follow-up time. Assuming the same participation rate as baseline, a final two-year follow-up of at least 1,200 participants is expected.

## Discussion

The experience of the BPRHS illustrates the challenges of conducting research in a Hispanic ethnic subgroup, and demonstrates the disparities faced by this community. The study has recruited 1,500 older adult Puerto Ricans, but considerable effort to obtain completed interviews has been required. Several studies have reported difficulties in recruiting minority research participants [65-68], especially Latino men [20]. It is possible that with door-to-door enumeration, women are more likely to be at home and therefore more frequently approached. They also appear to be more willing to participate than men, although this was not statistically significant in this study.

After enumeration, re-contacting individuals posed significant challenges. A key factor in the eventual success in locating individuals was recording information of close contacts. However, in many cases, field staff needed to return to the neighborhood to locate the individual. Recruitment completion was further delayed by frequent cancellations of appointments. These challenges led to loss of staff time and effort, and greatly increased the study cost over that originally estimated. Similarly, Eakin et al. reported the need to hire extra phone staff in order to increase retention of Latinos [21]. Interestingly, main reasons for cancellation included medical appointments and illness of the participant or a relative, which is consistent with the observed high prevalence of disease, and the sharing of burden among socially connected individuals.

Mistrust of scientific investigations is frequently reported as a major barrier to recruitment in minority participants [65]. Several strategies were used to facilitate recruitment, including employing a bilingual and ethnically diverse staff, and partnering with a local community organization. Recruitment at community events increased the study's visibility and involvement in the community, and enabled staff to obtain updated contact information for enrolled participants. Use of the media also reinforced the legitimacy of the study. Because the total community is relatively small, receptivity increased over time.

Efforts to keep the participants' trust and engagement in the study have helped sustain a high retention during two-year follow-up. As the study moves forward, questions on progression and mechanisms of diseases may be answered more accurately. Little is known about the environmental influences and life events of elderly Puerto Ricans living in the US; thus, possible cohort effects require further consideration. The wide age range of this group, constant migration patterns to/from Puerto Rico, limited social networks, and low residential stability may limit assumptions about cohort effects.

As with most epidemiological studies, selection bias could be operating in this study. For example, as those declining participation in the study were living in the US longer that those participating, possible selection bias by acculturation, which is highly correlated to years living in the US, may occur. Still, there was low acculturation in this sample, suggesting that such bias may not exist. The door-to-door recruitment method may have introduced selection bias, as participants recruited with this method, who comprised the majority of this cohort, had somewhat differing characteristics than those recruited through community events. The study followed exhaustive protocols to identify participants at home, making a great effort to expand recruitment with various strategies; the addition of participants from community approaches likely improved the representativeness for the study, as individuals who may have been seldom at home were included. Notably, the majority of Puerto Ricans aged 45-75 years identified by Census tracts, lived in neighborhoods and communities from which the study recruited.

Data from the 2000 Census show that, of Puerto Ricans aged 45-75 years living in high-density Hispanic blocks in Boston, 75% were 45-59 years, and 25% were 60-75 years [69]. Overall, the sample of this study was somewhat older, with about two-thirds in the younger and one-third in the older age range. When stratified by recruitment method, the age distribution more closely resembled that of the Census findings for those approached through flyers, community events or referrals (70% aged 45-59 years), whereas door-to-door recruitment yielded 60% in the younger category. Education levels for study participants were similar to those identified for Census data for Puerto Ricans in Boston; yet the study had fewer women in the older group that held a current job relative to 2000 Census data, while a greater number of men in the older age category reported working in this sample. This difference may be partly due to economic changes that have occurred since the year 2000. This sample of 1,500 individuals represents a fairly large proportion (15%) of the 10,241 Puerto Ricans in this age range living in the towns that we recruited from, as of 2000. Although this may suggest that a representative sample for this population was likely captured, the limited areas and approaches for recruitment may have reduced representativeness. Still, the results should be reasonably generalizable to similar communities of Puerto Rican adults living in high density urban areas in the US.

Hispanic subgroups are often combined together in health research; however, this practice may obfuscate important differences in subgroups. Though limited, accumulating studies provide evidence that health disparities differ considerably by subgroup [7,8,12-18,70]. The results of this study support observations that Puerto Ricans on the US experience considerable health disparities which exceed those reported for NHW or other Hispanic subgroups, including the more commonly studied Mexican Americans. The prevalence of physical and cognitive disability, type 2 diabetes, obesity, depressive symptomatology, hypertension, and self reported heart disease were higher in this sample, in relation to published reports for similarly-aged Mexican Americans [3,71-73]. Notably, the high prevalence of these conditions was observed even for those in the younger age category. For example, the prevalence of obesity in this sample of Puerto Rican men and women, aged 45-59 years, (43% and 60.5%, respectively) was higher than that reported by NHANES 2001-2004 for Mexican Americans in the same age range (36.3% and 52.2%, respectively) [74]. One caveat of this type of comparisons is that differences in survey methodology and year of data collection may affect the interpretation of the comparison.

## Conclusions

In conclusion, the BPRHS is the largest and most comprehensive study conducted to date on older adult Puerto Ricans living in the US, exclusively. It adds considerably to the efforts of other studies conducted in Hispanic elders, including the Health and Retirement Study, the Hispanic Established Populations for Epidemiologic Studies of the Elderly, and the Hispanic Community Health Study/Study of Latinos. This group experiences chronic health conditions at higher prevalence than those reported nationally for Mexican Americans or for NHW. The reasons for these health disparities remain largely unexplained, but illustrate the critical need for more research on the dynamics involved in these poor health outcomes. They also underscore the need to investigate Hispanic subgroups as unique cohorts, and to be careful when using the encompassing terms "Hispanic" or "Latino" when presenting health issues. The recruitment process portrays the challenges and opportunities involved in the enrollment of minority groups in epidemiological and clinical research, and may help strengthen future efforts for other studies. Understanding the unique needs of Puerto Ricans will inform interventions and public health practice, so that resources may be used prudently, and health disparities may be reduced. Improved scientific understanding of the etiology and progression of chronic conditions may also be attained.

## Abbreviations

ADL: Activities of Daily Living; BMI: Body mass index; BPRHS: Boston Puerto Rican Health Study; CESD: Center for Epidemiology Studies Depression scale; FFQ: Food frequency questionnaire; IADL: Instrumental Activities of Daily Living; MMSE: Mini mental state examination; NHANES: National Health and Nutrition Examination Survey; NHW: Non-Hispanic white; US: United States.

## Competing interests

The authors declare that they have no competing interests.

## Authors' contributions

KLT, the principal investigator, designed the study, directed its implementation, and supervised data analysis and interpretation; JMattei and SEN analyzed the data, interpreted the results, and contributed to writing the manuscript; BMC assisted with data analysis and the manuscript draft; JMendez coordinated the field activities and provided portions for the manuscript text; JN and JG collaborated with data analysis, interpretation of results, and portions of the text; JMO and LMF, co-principal investigators, helped design the study, conduct portions of its implementation, and interpret results. All authors reviewed the manuscript and approved the final version.

## Pre-publication history

The pre-publication history for this paper can be accessed here:

http://www.biomedcentral.com/1471-2458/10/107/prepub

## Supplementary Material

Additional file 1 **Detailed Methodology for the Boston Puerto RicanHealth Study**. A full description of the protocols used to obtain anthropometric and biochemical (blood, urine, saliva) measures. Click here for file

## References

[B1] Harris MI Hadden WC Knowler WC Bennett PH Prevalence of diabetes and impaired glucose tolerance and plasma glucose levels in U.S. population aged 20-74 yr Diabetes 1987 36 523 534 10.2337/diabetes.36.4.523 3817306

[B2] Harris MI Flegal KM Cowie CC Eberhardt MS Goldstein DE Little RR Wiedmeyer HM Byrd-Holt DD Prevalence of diabetes, impaired fasting glucose, and impaired glucose tolerance in U.S. adults. The Third National Health and Nutrition Examination Survey, 1988-1994 Diabetes Care 1998 21 4 518 524 10.2337/diacare.21.4.518 9571335

[B3] Ogden CL Carroll MD Curtin LR McDowell MA Tabak CJ Flegal KM Prevalence of overweight and obesity in the United States, 1999-2004 Journal of the American Medical Association 2006 295 13 1549 1555 10.1001/jama.295.13.1549 16595758

[B4] Ostchega Y Dillon CF Hughes JP Carroll M Yoon S Trends in hypertension prevalence, awareness, treatment, and control in older U.S. adults: data from the National Health and Nutrition Examination Survey 1988 to 2004 J Am Geriatr Soc 2007 55 7 1056 1065 10.1111/j.1532-5415.2007.01215.x 17608879

[B5] Saydah S Cowie C Eberhardt MS De Rekeneire N Narayan KM Race and ethnic differences in glycemic control among adults with diagnosed diabetes in the United States Ethn Dis 2007 17 3 529 535 17985509

[B6] U.S. Census Bureau, Population Division. National Population Projections http://www.census.gov/population/www/pop-profile/natproj.html

[B7] Barcelo A Gregg EW Pastor-Valero M Robles SC Waist circumference, BMI and the prevalence of self-reported diabetes among the elderly of the United States and six cities of Latin America and the Caribbean Diabetes Res Clin Pract 2007 78 3 418 427 10.1016/j.diabres.2007.06.008 17669541

[B8] Mainous AG Diaz VA Saxena S Geesey ME Heterogeneity in management of diabetes mellitus among Latino ethnic subgroups in the United States J Am Board Fam Med 2007 20 6 598 605 10.3122/jabfm.2007.06.070115 17954868

[B9] U.S. Census Bureau 2006. American Community Survey: ACS Demographic and Housing Estimates 2006 http://factfinder.census.gov/servlet/ADPTable?-geo_id=01000US&-qr_name=ACS_2006_EST_G00_DP5&-ds_name=ACS_2006_EST_G00_

[B10] Council on Scientific Affairs Hispanic Health in the United States JAMA 1991 265 2 248 252 10.1001/jama.265.2.248 1984156

[B11] Hajat A Lucas JB Kington R Health outcomes among Hispanic subgroups: data from the National Health Interview Survey, 1992-95 Adv Data 2000 310 1 14 10977762

[B12] Tucker KL Falcon LM Bianchi LA Cacho E Bermudez OI Self-reported prevalence and health correlates of functional limitation among Massachusetts elderly Puerto Ricans, Dominicans, and non-Hispanic white neighborhood comparison group J Gerontol A Biol Sci Med Sci 2000 55 2 M90 97 10737691 10.1093/gerona/55.2.m90

[B13] Falcon LM Tucker KL Prevalence and correlates of depressive symptoms among Hispanic elders in Massachusetts J Gerontol B Psychol Sci Soc Sci 2000 55 2 S108 116 10794195 10.1093/geronb/55.2.s108

[B14] Tucker KL Bermudez OI Castaneda C Type 2 diabetes is prevalent and poorly controlled among Hispanic elders of Caribbean origin Am J Public Health 2000 90 8 1288 1293 10.2105/AJPH.90.8.1288 10937011 PMC1446320

[B15] Bermudez OI Tucker KL Total and central obesity among elderly Hispanics and the association with Type 2 diabetes Obes Res 2001 9 8 443 451 10.1038/oby.2001.58 11500524

[B16] Chen H Bermudez OI Tucker KL Waist circumference and weight change are associated with disability among elderly Hispanics J Gerontol A Biol Sci Med Sci 2002 57 1 M19 25 11773208 10.1093/gerona/57.1.m19

[B17] Castaneda C Bermudez OI Tucker KL Protein nutritional status and function are associated with type 2 diabetes in Hispanic elders Am J Clin Nutr 2000 72 1 89 95 10871566 10.1093/ajcn/72.1.89

[B18] Lin H Bermudez OI Falcon LM Tucker KL Hypertension among Hispanic elders of a Caribbean origin in Massachusetts Ethn Dis 2002 12 4 499 507 12477135

[B19] Warnecke RB Oh A Breen N Gehlert S Paskett E Tucker KL Lurie N Rebbeck T Goodwin J Flack J Approaching health disparities from a population perspective: the National Institutes of Health Centers for Population Health and Health Disparities Am J Public Health 2008 98 9 1608 1615 10.2105/AJPH.2006.102525 18633099 PMC2509592

[B20] Rodriguez MD Rodriguez J Davis M Recruitment of first-generation Latinos in a rural community: the essential nature of personal contact Fam Process 2006 45 1 87 100 10.1111/j.1545-5300.2006.00082.x 16615255

[B21] Eakin EG Bull SS Riley K Reeves MM Gutierrez S McLaughlin P Recruitment and retention of Latinos in a primary care-based physical activity and diet trial: The Resources for Health study Health Educ Res 2007 22 3 361 371 10.1093/her/cyl095 16963726

[B22] Sheppard VB Cox LS Kanamori MJ Canar J Rodriguez Y Goodman M Pomeroy J Mandelblatt J Huerta EE Brief report: if you build it, they will come: methods for recruiting Latinos into cancer research J Gen Intern Med 2005 20 5 444 447 10.1111/j.1525-1497.2005.0083.x 15963169 PMC1490123

[B23] Chumlea WMC Guo SS Wholihan K Cockram D Kuczmarski RJ Johnson CL Stature prediction equations for elderly non-Hispanic white, non-Hispanic black, and Mexican-American persons developed from NHANES III data J Am Diet Assoc 1998 98 137 142 10.1016/S0002-8223(98)00036-4 12515412

[B24] Seeman TE Charpentier PA Berkman LF Tinetti ME Guralnik JM Albert M Blazer D Rowe JW Predicting changes in physical performance in a high-functioning elderly cohort: MacArthur studies of successful aging J Gerontol 1994 49 M97 M108 8169338 10.1093/geronj/49.3.m97

[B25] Dreon DM John EM DiCiccio Y Whittemore AS Use of NHANES Data to Assign Nutrient Densities to Food Groups in a Multiethnic Diet History Questionnaire Nutrition and Cancer 1993 20 223 230 10.1080/01635589309514290 8108272

[B26] McDowell M Briefel RR Warren RA Buzzard IM Feskanich D Gardner SN The Dietary Data Collection System - An Automated Interview and Coding System for NHANES III Fourteenth National Nutrient Databank Conference: June 19-21, 1989 1989 1989 Iowa City, IA 10

[B27] Delgado JL Johnson CL Roy I Trevino FM Hispanic Health and Nutrition Examination Survey: Methodological Consideration Am J Public Health 1990 80 Suppl 6 10 10.2105/AJPH.80.Suppl.6 9187575 PMC1404511

[B28] McDowell M Loria CM Cultural Considerations in Analyzing Dietary Data from the Hispanic Health and Nutrition Examination Survey National Nutrition Database Conference: 1989 1989 National Center for Health Statistics 43 46

[B29] Block G Subar AF Estimates of nutrient intake from a food frequency questionnaire: The 1987 National Health Interview Survey Journal of the American Dietetic Association 1992 92 8 969 977 1640041

[B30] U.S. Census Bureau, Housing and Household Economic Statistics Division: Poverty Thresholds http://www.census.gov/hhes/www/poverty/threshld.html

[B31] Bermudez OI Falcon LM Tucker KL Intake and food sources of macronutrients among older Hispanic adults: association with ethnicity, acculturation, and length of residence in the United States J Am Diet Assoc 2000 100 6 665 673 10.1016/S0002-8223(00)00195-4 10863569

[B32] Lin H Bermudez OI Tucker KL Dietary patterns of Hispanic elders are associated with acculturation and obesity J Nutr 2003 133 11 3651 3657 14608089 10.1093/jn/133.11.3651

[B33] Marin G Gamba RJ A New Measurement of Acculturation for Hispanics: The Bidimensional Acculturation Scale for Hispanics (BAS) Hispanic Journal of Behavioral Sciences 1996 18 3 297 316 10.1177/07399863960183002

[B34] Tropp LR Erkut S Coll CG Alarcon O Garcia HAV Psychological acculturation development of a new measure for Puerto Ricans on the U.S. mainland Educational & Psychological Measurement 1999 59 2 351 367 10.1177/00131649921969794 PMC3057082 21415932

[B35] Paffenbarger RS Jr Hyde RT Wing AL Lee IM Jung DL Kampert JB The association of changes in physical-activity level and other lifestyle characteristics with mortality among men N Engl J Med 1993 328 8 538 545 10.1056/NEJM199302253280804 8426621

[B36] Paffenbarger RS Wing AL Hyde RT Physical activity as an index of heart attack risk in college alumni Am J Epidemiology 1978 108 161 175 10.1093/oxfordjournals.aje.a112608 707484

[B37] Najjar MF Kuczmarski RJ Anthropometric data and prevalence of overweight for Hispanics: 1982-1984 1989 Hyattsville, Maryland: National Center for Health Statistics 2786659

[B38] Katz S Ford AB Moskowitz RW Jackson BA Jaffe MW Studies of illness in the aged. The index of ADL: A standardized measure of biological and psychosocial function JAMA 1963 185 12 914 919 14044222 10.1001/jama.1963.03060120024016

[B39] Seeman TE Charpentier P Berkman LF Predicting changes in physical performance in a high-functioning elderly cohort: MacArthur studies of successful aging J Gerontol 1994 49 M97 M108 8169338 10.1093/geronj/49.3.m97

[B40] Karno M Burnam MA Escobar JI L Hough R Eaton WW Development of the spanish-language version of the National Institute of Mental Health diagnostic interview schedule Arch Gen Phsychiatry 1983 40 1183 1188 10.1001/archpsyc.1983.01790100029003 6639287

[B41] Artiola Fortuny L Hermosillo Romo D Heaton RK Pardee RE III Eds Manual de Normas y Procedimientos para la Batería Neuropsicológica en Español 2000 Swets & Zeitlinger

[B42] Wolf-Klein G Silverstone F Levy A Brod M Screening for Alzheimer's disease by clock drawing Journal of the American Geriatrics Society 1989 37 8 730 734 2754158 10.1111/j.1532-5415.1989.tb02234.x

[B43] Beery K The Developmental Test of Visual-Motor Integration Manual, revised ed 1989 Cleveland: Modern Curriculum Press

[B44] Uhlmann RF Larson EB Effect of education on the mini-mental state examination as a screening test for dementia J Am Geriatr Soc 1991 39 9 876 880 1885862 10.1111/j.1532-5415.1991.tb04454.x

[B45] Mackinnon A McCallum J Andrews G Anderson I The Center for Epidemiological Studies Depression Scale in older community samples in Indonesia, North Korea, Myanmar, Sri Lanka, and Thailand J Gerontol: Psychological Sciences 1998 53B 6 P343 P352 10.1093/geronb/53b.6.p343 9826965

[B46] Miller TQ Markides KS Black SA The factor structure of the CES-D in two surveys of elderly Mexican Americans J Gerontol B Psychol Sci Soc Sci 1997 52 5 S259 269 9310098 10.1093/geronb/52b.5.s259

[B47] Moscicki EK Locke BZ Rae DS Boyd JH Depressive Symptoms Among Mexican Americans: The Hispanic Health and Nutrition Examination Survey Am J Epidemiol 1989 130 2 348 360 2750730 10.1093/oxfordjournals.aje.a115341

[B48] Radloff L The use of the Center for Epidemiological Studies - Depression Scale with older adults Clinical Gerontology 1986 5 119 136 10.1300/J018v05n01_06

[B49] Norbeck JS Modification of life event questionnaires for use with female respondents Res Nurs Health 1984 7 1 61 71 10.1002/nur.4770070110 6565302

[B50] Sarason IG Johnson JH Siegel JM Assessing the impact of life changes: development of the Life Experiences Survey J Consult Clin Psychol 1978 46 5 932 946 10.1037/0022-006X.46.5.932 701572

[B51] Norbeck JS Lindsey AM Carrieri VL The development of an instrument to measure social support Nurs Res 1981 30 5 264 269 10.1097/00006199-198109000-00003 7027185

[B52] Cohen S Kamarck T Mermelstein R A global measure of perceived stress J Health Soc Behav 1983 24 4 385 396 10.2307/2136404 6668417

[B53] Ramirez MT Hernandez RL Factor structure of the Perceived Stress Scale (PSS) in a sample from Mexico Span J Psychol 2007 10 1 199 206 17549893 10.1017/s1138741600006466

[B54] Remor E Psychometric properties of a European Spanish version of the Perceived Stress Scale (PSS) Span J Psychol 2006 9 1 86 93 16673626 10.1017/s1138741600006004

[B55] Tucker KL Bianchi LA Maras J Bermudez OI Adaptation of a food frequency questionnaire to assess diets of Puerto Rican and non-Hispanic adults Am J Epidemiol 1998 148 5 507 518 9737563 10.1093/oxfordjournals.aje.a009676

[B56] Bermudez OI Ribaya-Mercado JD Talegawkar SA Tucker KL Hispanic and non-Hispanic white elders from Massachusetts have different patterns of carotenoid intake and plasma concentrations J Nutr 2005 135 6 1496 1502 15930459 10.1093/jn/135.6.1496

[B57] Gao X Wilde PE Lichtenstein AH Bermudez OI Tucker KL The maximal amount of dietary alpha-tocopherol intake in U.S. adults (NHANES 2001-2002) J Nutr 2006 136 4 1021 1026 16549468 10.1093/jn/136.4.1021

[B58] Kwan LL Bermudez OI Tucker KL Low vitamin B-12 intake and status are more prevalent in Hispanic older adults of Caribbean origin than in neighborhood-matched non-Hispanic whites J Nutr 2002 132 7 2059 2064 12097693 10.1093/jn/132.7.2059

[B59] American Diabetes Association Diagnosis and classification of diabetes mellitus Diabetes Care 2006 29 Suppl 1 S43 48 16373932

[B60] Chobanian AV Bakris GL Black HR Cushman WC Green LA Izzo JL Jr Jones DW Materson BJ Oparil S Wright JT Jr The Seventh Report of the Joint National Committee on Prevention, Detection, Evaluation, and Treatment of High Blood Pressure: the JNC 7 report JAMA 2003 289 19 2560 2572 10.1001/jama.289.19.2560 12748199

[B61] The Practical Guide to the Identification, Evaluation and Treatment of Overweight and Obesity in Adults NIH Publication Number 00-4084 http://www.nhlbi.nih.gov/guidelines/obesity/prctgd_c.pdf

[B62] Radloff LS The CES-D Scale: A Self-Report Depression Scale for Research in the General Population Applied Psychological Measurement 1977 1 3 385 401 10.1177/014662167700100306

[B63] Mattei J Parnell L Lai CQ Garcia-Bailo B Adiconis X Shen J Arnett D Demissie S Tucker K Ordovas JM Disparities in allele frequencies and population differentiation for 101 disease-associated single nucleotide polymorphisms between Puerto Ricans and non-Hispanic whites BMC Genet 2009 14 10 45 10.1186/1471-2156-10-45 PMC2734553 19682384

[B64] Livak KJ Allelic discrimination using fluorogenic probes and the 5' nuclease assay Genet Anal 1999 14 5-6 143 149 10084106 10.1016/s1050-3862(98)00019-9

[B65] Yancey AK Ortega AN Kumanyika SK Effective recruitment and retention of minority research participants Annu Rev Public Health 2006 27 1 28 10.1146/annurev.publhealth.27.021405.102113 16533107

[B66] Levkoff S Sanchez H Lessons learned about minority recruitment and retention from the Centers on Minority Aging and Health Promotion Gerontologist 2003 43 1 18 26 12604742 10.1093/geront/43.1.18

[B67] Moreno-John G Gachie A Fleming CM Napoles-Springer A Mutran E Manson SM Perez-Stable EJ Ethnic minority older adults participating in clinical research: developing trust J Aging Health 2004 16 5 Suppl 93S 123S 10.1177/0898264304268151 15448289

[B68] Ness RB Nelson DB Kumanyika SK Grisso JA Evaluating minority recruitment into clinical studies: how good are the data? Ann Epidemiol 1997 7 7 472 478 10.1016/S1047-2797(97)00080-X 9349914

[B69] Bureau of the Census, US Department of Commerce: 2000 US Census Tables 2008 Washington, DC: Bureau of the Census

[B70] Allison MA Budoff MJ Wong ND Blumenthal RS Schreiner PJ Criqui MH Prevalence of and risk factors for subclinical cardiovascular disease in selected US Hispanic ethnic groups: the Multi-Ethnic Study of Atherosclerosis Am J Epidemiol 2008 167 8 962 969 10.1093/aje/kwm402 18283034 PMC4107279

[B71] Centers for Disease Control and Prevention National Center for Health Statistics Health Data Interactive. Diabetes, ages 20+, US, 1988-2006 (Source: NHANES) http://205.207.175.93/HDI/TableViewer/tableView.aspx?ReportId=61

[B72] Centers for Disease Control and Prevention National Center for Health Statistics. Health Data Interactive Chronic conditions, ages 18+: US, 1997-2008 (Source: NHIS) http://205.207.175.93/HDI/TableViewer/tableView.aspx?ReportId=101

[B73] Franzini L Ribble JC Keddie AM Understanding the Hispanic paradox Ethn Dis 2001 11 3 496 518 11572416

[B74] Centers for Disease Control and Prevention National Center for Health Statistics. Health Data Interactive Overweight/obesity, ages 20+: US, 1988-2006 (Source: NHANES) http://205.207.175.93/HDI/TableViewer/tableView.aspx?ReportId=76

