# Supplementary material for: The Boston Puerto Rican Health Study, a longitudinal cohort study on health disparities in Puerto Rican adults: challenges and opportunities
Source: BMC Public Health. 2010 Mar 1;10:107. doi: 10.1186/1471-2458-10-107 (PMC2848197; doi:10.1186/1471-2458-10-107)
Supplement: Additional file 1 — Detailed Methodology for the Boston Puerto RicanHealth Study. A full description of the protocols used to obtain anthropometric and biochemical (blood, urine, saliva) measures. [file 1471-2458-10-107-S1.DOC]

Additional File 1: Detailed Methodology for the Boston Puerto Rican Health Study.

*Anthropometric measures*

Standing height, knee height, weight, and waist and hip circumferences are measured in duplicate, following published techniques [1, 2]. Weight is measured using a clinical scale (Toledo Weight Plate, Model I5S, Bay State and Systems Inc. Burlington, MA). Height is measured with a SECA 214 Portable Stadiometer. Knee height is measured to estimate standing height for participants who are unable to stand or have a spinal curvature [1, 3].

Systolic and diastolic blood pressure are measured by trained interviewers using an electronic sphygmomanometer (Dinamap^TM^ Model 8260, Critikon, Tampa, FL) in duplicate, at three time points during the interview: once near the beginning, during the middle, and near the end of the interview, after short rests, where the subject is seated quietly. The second and third readings are averaged.

*Blood measures*

Participants are asked to fast for 12 hours prior to the blood draw. Blood samples are drawn in the home by a certified phlebotomist on the morning following the home interview.

A portable centrifuge is used in the home to immediately spin down the blood samples for analysis of vitamin C. Blood samples are carried back to the Nutrition Evaluation Laboratory (NEL) at the Human Nutrition Research Center on Aging on the day of collection in coolers equipped with dry ice. Blood samples are immediately cooled to 4°C and the plasma separated within 4 hours in a refrigerated centrifuge. The red blood cells are washed twice with cold saline.

Specimens are inventoried, checked against the roster, and logged into the NEL’s clinical information system according to standard operating procedures. Blood samples are prepared for the various analyses and sent for immediate analysis. Plasma aliquots are saved in 1 mL cryogenic, screw-cap tubes, and stored at -70°C for later processing. The following biomarkers are analyzed from blood:

1. Complete blood count (red blood cell count, white blood cell count, hematocrit, hemoglobin, mean corpuscular volume, platelet count, mean corpuscular hemoglobin and mean corpuscular hemoglobin concentration) are measured by electronic impedance, light scatter, double hydrodynamic sleeving system, in a HORIBA ABX Pentra C+ (ABX Diagnostics, Irvine, CA).
2. Serum glucose is measured using an enzymatic, kinetic reaction on the Olympus AU400e with Olympus Glucose Reagents (OSCR6121) (Olympus America Inc., Melville, NY).
3. Serum insulin is measured using the Immulite 1000 Insulin Kit (LKIN1) on the Immulite 1000 (Seimens Medical Solutions Diagnostics, Los Angeles, CA). This is a solid-phase, two-site chemiluminescent immunometric assay.
4. Glycosylated hemoglobin (HbA1c) is analyzed in a 2 step process where the final value is determined by a ratio of HbA1c to Hb along with a conversion factor. A whole blood hemolysate is analyzed by latex-enhanced immunoturbimetric to determine the HbA1c and by colorimetric, endpoint for hemoglobin, on the Cobas FARA using the Roche Unimate HbA1c kit (Roche Diagnostics, Indianapolis, Indiana).
5. Cholesterol is analyzed from the EDTA plasma used for the lipoprotein profile with an enzymatic endpoint reaction on the Olympus AU400e with Olympus Cholesterol Reagents (OSR6116) (Olympus America Inc., Melville, NY) [4].
6. High density lipoprotein (HDL) is analyzed using EDTA plasma with the enzymatic endpoint reaction on the Olympus AU400e with Olympus HDL Reagents (OSR6195). (Olympus America Inc., Melville, NY) [4, 5].
7. Triglycerides are obtained from the EDTA plasma used for the lipoprotein profile with an enzymatic endpoint reaction on the Olympus AU400e with Olympus Triglyceride Reagents (OSR6133) (Olympus America Inc., Melville, NY) [4, 6-8].
8. Low density lipoprotein (LDL) and very low density lipoprotein (VLDL) are calculated as: VLDL= tryglycerides/5; LDL= cholesterol – (VLDL + HDL).
9. Serum DHEA-S is measured using the Immulite 1000 DHEA-S Kit (LKDS1) on the Immulite 1000 (Seimens Medical Solutions Diagnostics, Los Angeles, CA). This is a solid-phase, two-site chemiluminescent immunometric assay.
10. Serum blood urea nitrogen (BUN) is measured using an enzymatic, kinetic reaction on the Olympus AU400e with Olympus BUN Reagents (OSR6134) (Olympus America Inc., Melville, NY) [9].
11. Serum creatinine is measured with a colorimetric, kinetic reaction on the Olympus AU400e with Olympus Creatinine Reagents (OSR6178) (Olympus America Inc., Melville, NY) [10-12].
12. Serum albumin is measured with a dye binding, endpoint reaction on the Olympus AU400e with Olympus Albumin Reagents (OSR6102) (Olympus America Inc., Melville, NY) [13].
13. Carotenoids are obtained from the serum using a colorimetric reaction with a Beckman DU640 Spectrophotometer (Beckman Coulter, Fullerton CA) [14].
14. Plasma pyridoxal-5'-phosphate (PLP) is determined enzymatically using tyrosine decarboxylase based on the principles described by Shin-Buehring et al. [15]. PLP activity is determined on the basis of release of tritiated tyramine following the incubation of tyrosine decarboxylase apoenzyme with the supernatant fraction of TCA-precipitated serum samples and tritium-labeled tyrosine. Pooled plasma is used for quality control. The radio-enzymatic reaction is ran on a Beckman LS 6500 Scintillation Counter (Beckman Coulter, Fullerton CA).
15. Plasma folate and vitamin B12 concentrations are determined by a radioassay method using a commercially available kit from Biorad. Pooled plasma is used for quality control. As of March 2007, the Bio-Rad has been discontinued. It has been replaced with the Immunoassay kits from Siemens Medical Solutions Diagnostics for use on the Immulite 1000. The matrix for the analysis of these measures will change from plasma to serum. Folate is analyzed using a competitive, liquid-phase ligand labeled protein chemiluminescent assay (LKFO1) and vitamin B12 is analyzed using a solid-phase, competitive chemiluminescent assay (LKVB1).
16. Total homocysteine in plasma is determined by a newly improved method based on the principles described by Araki and Sako [16]. This analysis employs a C18 column on a Waters HPLC instrument equipped with a WISP automatic injector and attached to a fluorimeter (Krato from Applied Sciences Inc.); and is detected with a Perkin Elmer 650-15 Fluorescence Spectrophotometer (Perkin Elmers., Waltham, MA)
17. Methyl Malonic Acid (MMA) is extracted from serum and derivatized to form a volatile compound, as described by Rasmussen [17]. This derivative is analyzed by GC/MS and quantified based on the amount of a deuterated form of MMA (D3) added to the samples prior to extraction. Analysis is done on a Hewlett-Packard 5791A (Hewlett-Packard, Houston, TX) and dried on a Techne's Dri-block (Bibby Scientific, Burlington, NJ).
18. Ascorbate (vitamin C) is analyzed by paired-ion, reversed-phase IAPLC coupled with electrochemical detection. Samples are injected with an autosampler, (1100 series, Hewlett Packard). Ascorbate is detected at an applied potential of +0.6 V by a LC 4B amperometric electrochemical detector (Bioanalytical Systems, West Lafayette, IN). Ascorbate elutes as a single peak with a retention time of 5.5 min. Peaks are integrated with a ChemStation (Hewlett Packard). Ascorbate concentration is calculated based on a calibration curve, and its concentration expressed in μMol/L [18].
19. 25-hydroxy vitamin D is obtained from plasma with a competitive protein binding reaction using an LKB Wallac Rackbeta 1215 Counter (Perkin Elmers, Waltham MA) and Packard COBRA Software [19, 20].
20. High sensitivity C-reactive protein is measured in serum, using the Immulite 1000 High Sensitive CRP Kit (LKCRP1) on the Immulite 1000 (Seimens Medical Solutions Diagnostics, Los Angeles, CA). This is a solid-phase, two-site chemiluminescent immunometric assay.

Urinary measures

A 12-hour urine sample is collected the morning after the home interview. The 12-hour period begins on the evening after the home interview and ends the next morning as determined by the subject’s work and normal eating schedules. Urine samples are acid stabilized. Cortisol is analyzed with direct immunoenzymatic colorimetric method with an ALPCO cortisol assay (ALPCO, Windham, NH), and epinephrine and norepinephrine with 2-CAT enzyme immunoassay (Rocky Mountain Diagnostics, Colorado Springs, CO), and read on a Dynex MRX 96-well plate reader (Dynex Technologies**,** Chantilly, VA). The three measures are standardized by multiplying each measure by total urine volume and dividing by urinary creatinine excretion.

*Salivary measures*

Two samples are collected from each participant (evening specimen and morning specimen – 12 hours apart). Each sample is collected in a 2 ml cryovial. Participants use a plastic straw to collect the saliva inside the cryovial provided. They are instructed that chewing on a plastic straw can be utilized as a salivary stimulant and is particularly useful to stimulate the saliva volume collected. The sample collected in the evening by is stored inside their freezer at –20ºC. The saliva sample collected by the phlebotomist the following morning is kept in ice at 4ºC until deliver to the NEL. Upon arrival into the NEL, the specimens are immediately stored at -80ºC. Salivary cortisol is analyzed with an expanded range, high sensitivity enzyme immunoassay kit (Salimetrics, Inc, State College, PA), and read on a Dynex MRX 96-well plate reader (Dynex Technologies**,** Chantilly, VA).

*References*

1. Chumlea WMC, Guo SS, Wholihan K, Cockram D, Kuczmarski RJ, Johnson CL: **Stature prediction equations for elderly non-Hispanic white, non-Hispanic black, and Mexican -American persons developed from NHANES III data.** *J Am Diet Assoc* 1998, **98**:137-142.

2. Najjar MF, Kuczmarski RJ: **Anthropometric data and prevalence of overweight for Hispanics: 1982-1984**. Hyattsville, Maryland: National Center for Health Statistics; 1989.

3. Bermudez OI, Becker EK, Tucker KL: **Development of sex-specific equations for estimating stature of frail elderly Hispanics living in the northeastern United States**. *Am J Clin Nutr* 1999, **69**(5):992-998.

4. Allain CC, Poon LS, Chan CS, Richmond W, Fu PC: **Enzymatic determination of total serum cholesterol**. *Clin Chem* 1974, **20**(4):470-475.

5. Warnick G, Benderson J, Albers J: **Dextran sulfate-Mg2+ precipitation procedure for quantitation of high-density-lipoprotein cholesterol**. *Clin Chem* 1982, **28**:1379-1388.

6. Trinder P: *Ann Clin Biochem* 1969, **6**:24-27.

7. Spayd RW, Bruschi B, Burdick BA, Dappen GM, Eikenberry JN, Esders TW, Figueras J, Goodhue CT, LaRossa DD, Nelson RW *et al*: **Multilayer film elements for clinical analysis: applications to representative chemical determinations**. *Clin Chem* 1978, **24**(8):1343-1350.

8. Esders TW, Michrina CA: **Purification and properties of L-alpha-glycerophosphate oxidase from Streptococcus faecium ATCC 12755**. *J Biol Chem* 1979, **254**(8):2710-2715.

9. Henry JB: **Clinical diagnosis and management by laboratory methods**. In*.* Philadelphia: Saunders; 1974: p.260.

10. Jaffe M: **Uber den Niederschlag welchen Pikrinaure in normalem Harn erzeugt and iiber eine neueReaktion des Kreatinins.** *Z Physio Chem* 1886, **10**:391.

11. Larsen K: **Creatinine assay by a reaction-kinetic principle**. *Clin Chim Acta* 1972, **41**:209-217.

12. Tietz NW: **Fundamentals of Clinical Chemistry**. In*.* Philadelphia: Saunders; 1987: p. 673-684.

13. Doumas BT, Watson WA, Biggs HG: **Roche diagnostic systems Inc procedure number 44902, as modified in 1985**. *Clin Chem Acta* 1971, **31**:87-90.

14. Roels OA, Trout, M., Lui, N.S.T., Anderson, O.R.: **Vitamins and Hormones: Vitamin A and Carotene**, vol. 7. New York: Academic Press; 1972.

15. Shin-Buehring Y, Rasshofer R, Endres W: **A new enzymatic method for pyridoxal-5'-phosphate determination.** *Journal of Inherited Metabolic Disorders* 1981, **4**:123-124.

16. Araki A, Sako Y: **Determination of free and total homocysteine in human plasma by high-performance liquid chromatography with fluorescence detection**. *J Chromatography* 1987, **422**:43-52.

17. Rasmussen K: **Solid-phase sample extraction for rapid determination of methylmalonic acid in serum and urine by a stable-isotope-dilution method**. *Clin Chem* 1989, **35**(2):260-264.

18. Martin A, Frei B: **Both intracellular and Extracellular vitamin C inhibit atherogenic modification of LDL by human vascular endothelial cells.** *Arterioscler Thromb Vasc Biol* 1997, **17**:1583-1590.

19. Chen TC, Turner, A.K., and Holick, M.F.: **Methods for the determination of the circulating concentration of 25-hydroxyvitamin D**. *J Nutr Biochem* 1990, **1**:315-319.

20. Mayer E, Schmidt-Gayk, H.: **Interlaboratory comparison of 25-hydroxyvitamin D determination**. *Clin Chem* 1984, **30**:1199-1204.
